# Study on the Diffusion Characteristics of Polymer Grouting Materials Applied for Crack Filling in Underground Mines Based on Numerical Simulation and Experimental Methods

**DOI:** 10.3390/polym16182612

**Published:** 2024-09-15

**Authors:** Xuanning Zhang, Ende Wang

**Affiliations:** Department of Geology, College of Resources and Civil Engineering, Northeastern University, Shenyang 110819, China; 1910354@stu.neu.edu.cn

**Keywords:** polymer, crack filling, hydrodynamics, numerical simulation, diffusion law

## Abstract

Polymer grouting materials are increasingly used in the filling of mine fissures. Unlike conventional inorganic grouting materials, the self-expansion of polymers adds complexity to their diffusion process within the crack. The objective of this research was to examine how polymer grouting material spreads in cracks at ambient temperatures and pressure. The investigation involved conducting grouting tests and performing numerical fluid simulation calculations using the finite-volume method in the computational fluid dynamics software, ANSYS FLUENT 2022 R1. The fluid volume approach was employed to determine the boundary between fluid and air and to ascertain the variation patterns of density in the slurry and the fracture system. This study applied the principles of fluid mechanics to investigate the patterns of variation in the physical characteristics of polymer grouting materials, including their density, pressure, flow velocity, and movement distance, during the diffusion process. The results indicated that the density of the polymer grouting material decreased exponentially over time throughout the diffusion process. With the increase in the grouting’s volume, the grout’s pressure and the permeable distance of the grout increased. The slurry’s pressure near the grouting hole exceeded the other points’ pressure. The physical parameters of the slurry were numerically simulated by ANSYS FLUENT 2022 R1 software, and the results were compared with the experimental data. After comparing the numerical simulation results with the test data, it was clear that the numerical simulation method was superior in accurately predicting the distribution pattern of each parameter of the polymer slurry during diffusion. The grouting volume, pressure distribution, and real-time change in the position of the flow of slurry could be efficiently determined through numerical calculation and simulated grouting tests. This work can offer valuable information for designing polymer grouting materials used in underground mine fissures.

## 1. Introduction

Fissures in rocks during mining compromise the rock’s integrity, accelerate weathering, increase water permeability, and consequently weaken the rock’s strength and stability. As mining operations go deeper into metal mines, the geological conditions become more intricate and diverse [1,2,3,4]. The intersections of fissures within the rock formations grow more complex, leading to frequent occurrences of sudden water surges [5,6]. The sudden increases in activity present a notable danger to both the safety of workers on site and the efficiency of the mines’ operations. The safety and productivity of mining operations are at significant risk due to these factors affecting construction workers’ well-being. Grouting technology is a crucial method for managing underground mine disasters and is one of the primary techniques used for water plugging, reinforcement, and fissure filling [7,8]. Its role in controlling seepage and strengthening engineering structures is indispensable [9,10]. This technology has been extensively applied in various engineering fields such as water conservancy, transportation, and mining [11]. It is crucial for the safety of mine projects, especially those carried out below the surface [12].

In a project to fill fissures in underground mines, the type of grouting material chosen for use directly determines the advantages and disadvantages of the grouting’s effect [13]. The permeability of traditional grouting reinforcement materials such as cement and clay is restricted by their large particle sizes, hindering their ability to effectively fill and heal the smaller cracks in the rock formations found in deep underground mines [14,15,16]. This is a problem caused by fine fissures and groundwater seepage [17]. They exhibit weak bonding capabilities, making it difficult to manage the gel’s duration precisely. Additionally, they readily disperse when exposed to water, compromising their use in challenging conditions [18,19]. However, polyurethane slurry, a lightweight organic polymer grouting material, can be more successfully used to address the problems of water plugging and reinforcement in mining projects due to its excellent water impact resistance, short gel curing time, non-toxicity, and environmental friendliness [20]. It also has a high expansion rate, high permeability, and durability [21]. Thus, while traditional inorganic grouting materials’ applicability has been restricted by historically relevant factors, polymer chemical grouting materials, such as polyurethane, have drawn more attention from researchers and are frequently utilized in underground mine projects [22,23,24]. Engineers have come to appreciate polyurethane’s quick water plugging, rapid reinforcement, and ability to prevent leaks.

The ability of the surrounding medium to confine the material, the reaction time, and the ambient pressure are all connected to the swelling properties of polymer materials. Polymers in cracks have a more complex diffusion mechanism than inorganic non-expansive grouting materials. Simulated fissure grouting experiments and numerical simulations are valuable tools for studying the mechanism of slurry’s dispersion because grouting is a hidden project. In recent years, numerous researchers have conducted extensive research in this field. Liu et al. [25] examined the impact of slurry’s parameters, the fissures’ size, and the grid’s size on the efficacy of fissure grouting by numerically simulating the grouting of regular and monolithic fissure networks. The impact of rock bodies’ fluid–solid interaction on the grouting’s performance was recognized by Yan et al. [26], who conducted a study on the evolution of rock fissures during the grouting process and created a numerical simulation of two-dimensional grouting in rock fissures, taking fluid–solid coupling into consideration. Based on a one-dimensional channel model, Hassler et al. [27] created a fissure model and then simulated and examined the slurry’s diffusion within the fissure. Li et al. [28] created a quasi-3D model of planar fissure grouting and, by taking into account the influence of the fissure opening, reduced the modeling complexity and transformed the 3D problem into a 2D problem. A computational model of fracture grouting was developed by Saeidi et al. [29], who also examined the impact of the fracture opening and other variables on the slurry’s diffusion performance within the fracture. Yang et al. [30] generated a random fracture network using the Monte Carlo approach and examined the impact of mid-fracture deformation on the grouting during the grouting process, as well as the distribution of parameters including pressure and the slurry’s flow rate. Li et al. [31] proposed a radial diffusion model for a known variable-density self-expanding grouting material in a planar fracture based on the theory of viscous fluid dynamics. Considering the effects of the initial filling radius, crack width, and time on grouting diffusion, the analytical solutions of the grouting’s diffusion radius and pressure distribution were derived. The results showed that the diffusion characteristics of grout are closely related to the evolution law of the density, as when the grout’s density was 1.25 g/cm^3^, the maximum diffusion radius of the grout reached 0.6 m within 30 s, and the grout’s pressure in the center of the grouting hole could reach 10 kPa. The diffusion behavior of grouting materials in the fissures has been better understood because of this research. Alternatively, utilizing numerical modeling techniques along with simulated fissure grouting experiments is essential for studying the dispersion process of a polymer slurry, considering the changing density of the polymer slurry over time and the self-expanding characteristic of polymer. There have not been many published studies in this field, though.

In this research, polyurethane slurry was classified as a Newtonian liquid with a fluctuating density. A model was built to analyze how the density changes over time, and the pattern of the change in density over time in polyurethane slurry was determined through indoor testing. ANSYS FLUENT 2022 R1 software was used to numerically analyze the diffusion process of polyurethane slurry in the cracks. The calculated results of the numerical analysis were validated by simulated grouting tests, and parameters such as pressure, the flow’s velocity, and movement distance were among the parameters obtained by solving the control equations. This work contributes to the advancement of polyurethane grouting theory and offers recommendations for its use in filling mine fissures.

## 2. Numerical Models and Experimental Methods

### 2.1. The Polymer Slurry’s Diffusion Process

The movement process of polyurethane slurry within cracks can be divided into three stages: the static pressure injection stage, the slurry’s expansion stage, and the curing stage.

In the static injection phase, the density and viscosity of the polyurethane slurry remain largely constant, with the flow primarily governed by the injection pressure. Following a period of injection, the grouting’s pressure ceases, and the chemical reaction commences gradually. As the slurry transitions into the expansion phase, its density and viscosity start to change. The flow field pressure of the polyurethane slurry increases due to the self-expansion force induced by the chemical reaction, facilitating further penetration into the crack. Concurrently, the chemical reaction within the slurry generates a significant amount of carbon dioxide gas, which expands the volume of bubbles within the material and leads to a reduction in its density. With the progression of the chemical reaction, the polyurethane mixture transitions from a liquid state to a viscous semi-solid substance, culminating in the gradual cessation of the slurry’s flow.

In the curing stage, the polyurethane mixture stops expanding and spreading. The response continues to advance through the process of cross-linking. At this stage, the strength and chemical stability of the polyurethane-solidified body is further improved.

### 2.2. Fundamental Assumption

The following assumptions are made about the diffusion process of a polyurethane slurry in a fracture.

The polyurethane grout is a slightly compressible Newtonian fluid, and the grout is homogeneous. The change in energy during the slurry’s movement was ignored. The change in the viscosity of the slurry before gelation was neglected. The spread of the mixture was a smooth, ongoing movement, and the speed of the mixture at the point where the fracture surface met the mixture was zero. The fractured surface was rigid and did not deform.

### 2.3. Numerical Computation Method

After the polyurethane slurry has been filled into the fracture, the slurry is in contact with the gas in the fracture, and the two-phase interface between the gas and the liquid changes with the movement of the slurry. For the flow–diffusion process of the polymer slurry in the fracture, the VOF method was introduced to describe the two-phase distribution of the medium. The YOF method, known as the Volume of Fluid Method, is a numerical computational method used to simulate and analyze fluid dynamics problems. The fundamental concept involves analyzing the ratio function f of the fluid’s volume to the grid’s volume within a grid cell to track changes in the fluid and determine the free surface. Instead of tracking the motion of a mass on the free surface, this method models the behavior of the fluid on the basis of the concept of the fluid’s volume fraction. By monitoring how the volume fraction changes over time, it is possible to follow the shape and location of the interface.

In the VOF model, the computational region is discretized into a series of small grid cells, each of which has an internal volume fraction that represents the proportion of fluid occupying that cell. The value of the volume fraction is equal to the ratio of the volume of fluid within a cell to the volume of that grid cell. According to the value of the volume fraction, the state of the cell can be determined as shown in Equation (1).

(1) $$F(X,t)=\frac{V_{p}}{V}$$

In Equation (1), X denotes the vector coordinates of the grid node, V denotes the volume of the grid cell, V_p_ denotes the volume of the polymer slurry in the grid cell, and F denotes the fluid volume fraction. When the value of F is equal to 0, it means that this grid cell is all gas, and no slurry is present. When the value of F is in the range of 0 to 1, it indicates that there is both gas and slurry in the grid cell, and there is a gas–liquid interface. When the value of F is equal to 1, it means that the space within this grid cell is completely filled with slurry, and no gas is present.

### 2.4. Governing Equations

#### 2.4.1. Continuity Equation

To ensure that the mass conservation law is met, the movement of polymer slurry in the crack must adhere to Euler’s method. This involves analyzing the fluid’s motion by selecting a control body or microelement within the fluid-filled space. The focus is on the fluid within this control body or microelement, where the increase in mass over time equals the mass of the net inflow of fluid during that interval. The continuity equation is shown in Equation (2).

(2) $$\frac{\partial \rho }{\partial t}+\frac{\partial (\rho u)}{\partial x}+\frac{\partial (\rho v)}{\partial y}+\frac{\partial (\rho w)}{\partial z}=0$$

Equation (2) includes the polymer density denoted by ρ, time denoted by t, and the velocity components u, v, and ω on the x-, y-, and z-axes, respectively. In order to simplify the equation, the Hamiltonian operator ▽ is introduced, as shown in Equation (3). The continuity equation can be rewritten as shown in Equation (4), where U represents the velocity vector.

(3) $$\nabla =\frac{\partial }{\partial x}\vec{i}+\frac{\partial }{\partial y}\vec{j}+\frac{\partial }{\partial z}\vec{k}$$

(4) $$\frac{\partial \rho }{\partial t}+\nabla \cdot (\rho U)=0$$

During the flow of the polymer slurry, due to the conservation of mass throughout the fluid, the volume fraction of the fluid in the grid changes with time after the fluid enters the cell grid and comes into contact with the air in the VOF model, so the volume fraction of the slurry must satisfy the convection equation, as shown in Equation (5).

(5) $$\frac{\partial F}{\partial t}=-U\cdot \nabla F$$

#### 2.4.2. Momentum Equation

According to Euler’s method for fluid microelements, and in accordance with Newton’s second law, the rate of increase in momentum of the fluid in a microelement is equal to the sum of various forces acting on the microelement. The fluid’s motion process must satisfy the conservation of momentum as shown in Equation (6).

(6) $$\frac{\partial (\rho U)}{\partial t}+\nabla \cdot (\rho UU)=-\nabla p+\nabla \cdot [\mu (\nabla U+\nabla U^{T})]+\rho g$$

In this equation, ρ denotes the weighted density of the fluid in the control body, and μ denotes the weighted viscosity of the fluid in the control body. F_a_ is taken to denote the volume fraction of air in the control body, and (1 − F_a_) denotes the volume fraction of the polymer slurry in the control body. Then the weighted density and weighted viscosity can be expressed by Equations (7) and (8), where ρ_a_ and ρ_p_ denote the air’s density and the polymer’s density, respectively; and μ_p_ and μ_p_ denote the air’s viscosity and the polymer’s viscosity, respectively.

(7) $$\rho =F_{a}\rho_{a}+\left(1-F_{a}\right)\rho_{P}$$

(8) $$\mu =F_{a}\mu_{a}+\left(1-F_{a}\right)\mu_{P}$$

### 2.5. Density Function

It can be seen from the continuity equation and the momentum equation that the density of polyurethane slurry is an important parameter for solving the control equation. In order to obtain the change rule of the density of polyurethane slurry with time, in this study, the variation in the volume of grout was measured by laboratory tests. A certain amount of slurry was placed in the measuring cylinder, the change rule of the volume of polyurethane with time was measured through a test of the change in the expansion of the polyurethane slurry in a measuring cylinder, and the change curve of the density of the slurry with time could be obtained because the mass of the slurry was always the same. The specific test steps were as follows.

1. Fill a cylindrical container with a graduated scale with prepared homogeneous, uncured polyurethane.
2. Record the time and volume at this point and start the timer. At the same time, make sure that the ambient temperature and humidity remain constant during the test.
3. Record the volume of the slurry at 5 s intervals until the slurry is completely set, the volume no longer changes, and the test is stopped.

**Figure 1:**
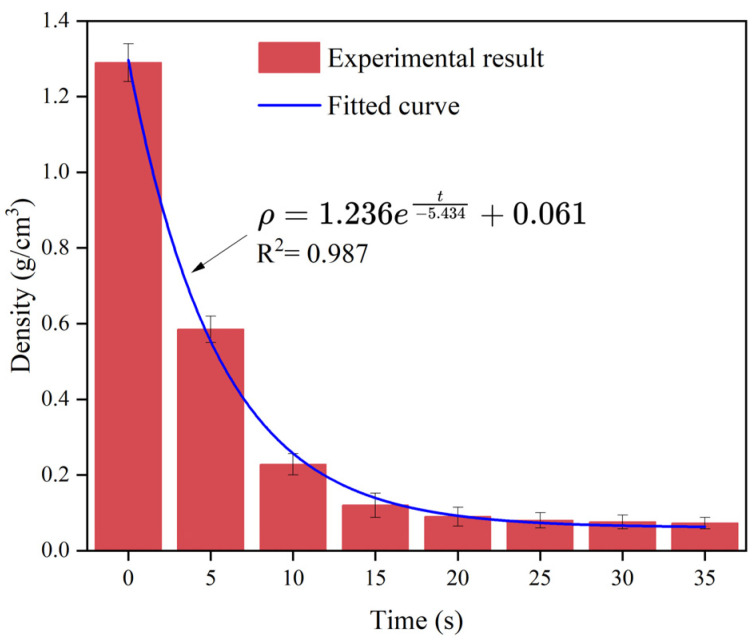
The variation law of the polymer’s density over time.

Figure 1 displays the evolution of the polyurethane slurry’s density over time under ambient temperature and pressure conditions indoors. Figure 1 illustrates that the polyurethane slurry started with a density of approximately 1.29 g/cm^3^, which then decreased exponentially as the slurry foamed and expanded. Within 20 s, the density dropped to 0.09 g/cm^3^ and eventually stabilized at around 0.073 g/cm^3^ after 35 s.

### 2.6. Simulated Crack Grouting Test

**Figure 2:**
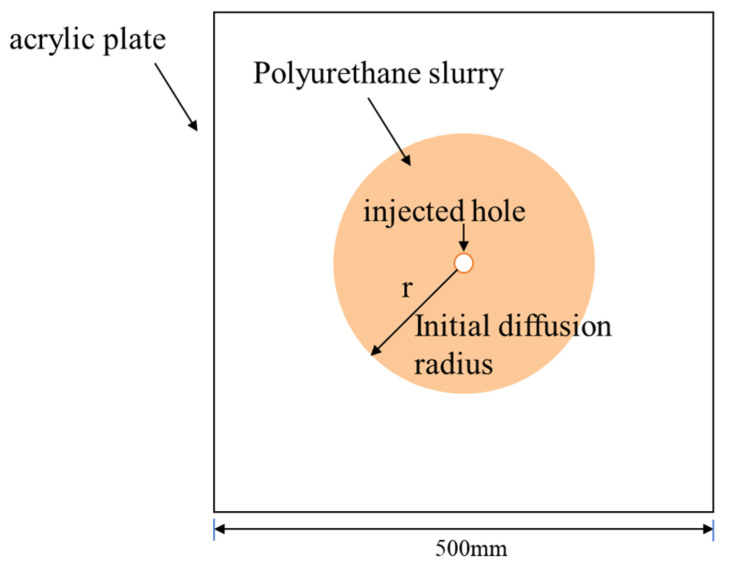
Top view of polyurethane in a simulated crack.

The fissure’s design included a pair of 500 mm square acrylic plates, with the gap between them being modified using padding. The polymer was transfused via a tiny opening positioned at the middle of the panels to monitor the leakage of the polymer in the simulated crack, as depicted in Figure 2; furthermore, the sensors were used to track the variations in pressure while the polyurethane mixture seeped in.

## 3. Numeric Calculations

### 3.1. Discretization

Discretizing the governing equations was achieved through utilization of the finite volume technique. To solve this problem numerically, the direct application of each equation required the development of a specialized computational procedure, which not only complicated the computational process but also had relatively low generalizability. A generalized form of the control equations not only made it easier to develop the corresponding computational procedures but also improved the ability and efficiency of solving various problematic situations. Therefore, the establishment of a generalized form of control equations facilitated the construction of algorithms, the analysis of problems, and the calculation of solutions in a more efficient manner.

For the control body, the differential form of the generalized control equation for variable parameters is shown in Equation (9)

(9) $$\underset{\mathrm{Transient}\;\mathrm{term}}{\underset{︸}{\frac{\partial (\rho \phi )}{\partial t}}}+\underset{\mathrm{Convective}\;\mathrm{term}}{\underset{︸}{\nabla \cdot (\rho U\phi )}}=\underset{\mathrm{Diffusion}\;\mathrm{term}}{\underset{︸}{\nabla \cdot (\Gamma^{\phi }\nabla \phi )}}+\underset{\mathrm{Source}\;\mathrm{item}}{\underset{︸}{Q^{\phi }}}$$

where ϕ denotes the generic variable parameter, which could be a variable such as velocity, temperature, etc., in this study; ϕ refers to the speed U. The generalized dispersion coefficient Γ represents the viscosity coefficient μ. The mechanical influence is represented by the source term Q^ϕ^.

According to the physical meaning of each item in the Navistox equation, the Navistox equation is divided into the following terms: the transient term, the convection term, the diffusion term, and the source term. The transient term describes the change in a fluid with respect to time and is usually represented as the partial derivative of the velocity field with respect to time. The convection term describes the transport of matter by a fluid due to its own motion, and the convection term reflects the relationship between velocity and changes in position in the fluid. The diffusion term, also known as the viscous term, describes the transfer of momentum within a fluid due to viscous action. The diffusion term is usually related to the coefficient of viscosity and the velocity gradient. This term is a manifestation of the internal friction force of the fluid, which causes the differences in the velocity of the fluid to become uniform. The source term represents the influence of external forces on the fluid’s motion.

The generalized control equations were discretized using the finite volume method. Firstly, the transient form of the fluid motion was considered, and the integral form of the generalized control equation in the transient form was obtained as shown in Equation (10).

(10) $$\oint_{V_{C}}\nabla \cdot (\rho U\phi )dV=\oint_{V_{C}}\nabla \cdot \left(\Gamma^{\phi }\nabla \phi \right)dV+\int_{V_{C}}Q^{\phi }dV$$

According to the Gaussian dispersion theorem, the area integrals of the diffusion and convection terms were replaced by the volume integrals, as shown in Equation (11).

(11) $$\oint_{\partial V_{C}}\left(\rho U\phi \right)\cdot d\vec{S}-\oint_{\partial V_{C}}\left(\Gamma^{\phi }\nabla \phi \right)\cdot d\vec{S}=\int_{V_{C}}Q^{\phi }dV$$

The area integrals of the convective and diffusive terms were expanded to obtain the general discretization equations for the convective and diffusive terms as follows, with Equation (12) denoting the general discretization equation for the convective term and Equation (13) denoting the general discretization equation for the diffusive term.

(12) $$\oint_{\partial V_{C}}\left(\rho U\phi \right)\cdot d\vec{S}=\sum_{f\sim \mathrm{faces}(V_{C})}\left(\int_{f}\left(\rho U\phi \right)\cdot d\vec{S}\right)$$

(13) $$\oint_{\partial V_{C}}\left(\Gamma^{\phi }\nabla \phi \right)\cdot d\vec{S}=\sum_{f\sim \mathrm{faces}(V_{C})}\left(\int_{f}\left(\Gamma^{\phi }\nabla \phi \right)\cdot d\vec{S}\right)$$

Equation (14) was derived by applying the Gaussian integration law to calculate the volume integral of the source term. In this calculation, ip represents the integration point, ip(f) indicates the number of integration points across the area, and ω_ip_ is the weight function. As only one integration point was considered for each control microelement, the value of ω_ip_ was set to 1.

(14) $$\int_{V}Q^{\phi }dV=\sum_{\mathrm{ip}\sim \mathrm{ip}(f)}\left(Q_{\mathrm{ip}}^{\phi }\omega_{\mathrm{ip}}V\right)=Q_{C}^{\phi }V_{C}$$

**Figure 3:**
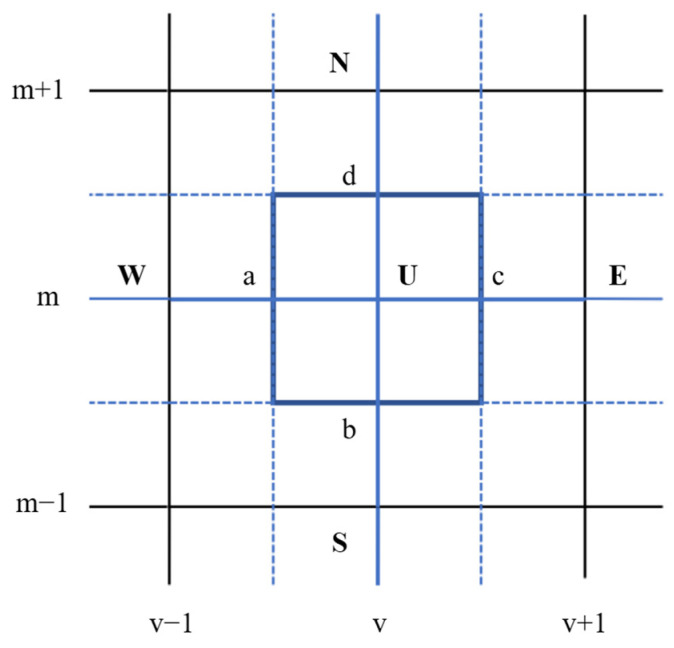
Schematic diagram of the two-dimensional control body’s mesh.

In the numerical computation process, this study used a pressure-based solver to solve the structural mesh. Figure 3 illustrates a schematic diagram of a two-dimensional control volume discretized with a structured grid. The velocity components u and v, as well as the pressure p, are all computed within the control volume U. The labels a, b, c, and d denote the corresponding four boundary surfaces of the control volume U. The directions N, W, S, and E, respectively, indicate the four control volumes adjacent to the control volume U. The SIMPLE algorithm was used to compute the velocity vector and the pressure field, and a first-order implicit integral was used to discretize the time, setting the time step to 0.01 s. The Courant number is a dimensionless parameter used in numerical simulations to determine whether a physical process in a calculation is stable. The Courant number is defined as the ratio of the time step to the maximum stable time step determined by the mesh size and the fluid’s velocity. In general, as the Courant number changes from small to large, the speed of convergence gradually increases, but the stability gradually decreases. According to implicit integral solving, the Courant number for this study was 5.

### 3.2. Initial and Boundary Conditions

**Figure 4:**
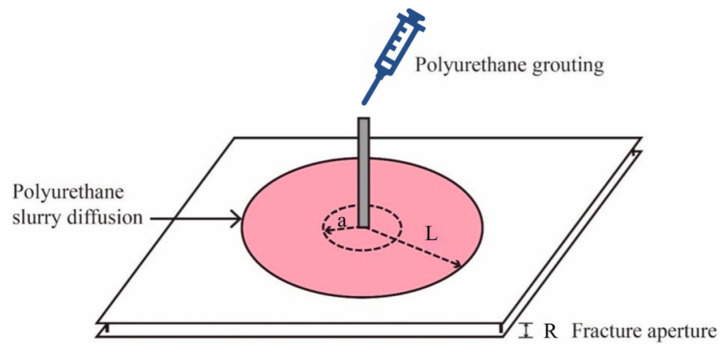
Schematic diagram of the diffusion process of polymer slurry.

This research involved a numerical model simulating the spread and dispersion of a polymer slurry within a solitary crevice, with the region of the fissure having a side length of 500 mm and a pore diameter denoted as R. To accurately replicate the slurry’s diffusion under standard atmospheric conditions, the study specified a surface pressure of zero at the fissure’s outlet and assumed no slippage at the upper and lower walls of the crevice. Figure 4 illustrates the diffusion of the polymer slurry, with ‘a’ indicating the distance of the flow during the initial penetration stage before the polyurethane slurry expanded, and ‘L’ indicating the distance of movement after expansion. The numerical simulation phase began after the completion of the hydrostatic dispersion of the slurry. Equation (15) demonstrates the connection between the initial distance of the slurry’s flow (a), the mass of the grout (Q), the fissure’s aperture (R), and the initial density of the slurry (ρ_0_) in the hydrostatic phase where the slurry’s mass remained relatively constant. The initial velocity and pressure of the slurry were set to zero, the density as a function of time was as shown in Figure 1, and the viscosity of the slurry was set to 1.0 Pa·s. In addition, in the process of simulating crack grouting, the boundary pressure of the crack and the slurry’s flow velocity at the boundary were set to zero on free surfaces.

(15) $$Q=\rho_{0}\pi a^{2}R\to a=\sqrt{\frac{Q}{\pi \rho_{0}R}}$$

The essence of SIMPLE algorithm is a “guess–correction” process of the velocity field and pressure field, and the convergent solution is obtained by iterative solution. The main process of the numerical calculation was as follows: (1) initializing each unit’s fluid volume function and setting the boundary conditions; (2) initializing the pressure and velocity vectors; (3) according to the time and fluid volume function, determining the characteristic parameters of each unit of fluid material, such as density; (4) obtaining the current velocity by solving the discrete equation of momentum; (5) solving the pressure equation to obtain the value of pressure; (6) updating the current flow velocity and values of pressure; (7) repeating Steps (4) to (6) until the flow field converges; (8) solving and updating the fluid volume function, and constructing the moving interface; and (9) returning to Step (2), updating the time, and starting a new round of solving.

## 4. Results and Discussion

### 4.1. Distribution of the Pressure Field 

#### 4.1.1. Results of the Numerical Calculation of Pressure 

**Figure 5:**
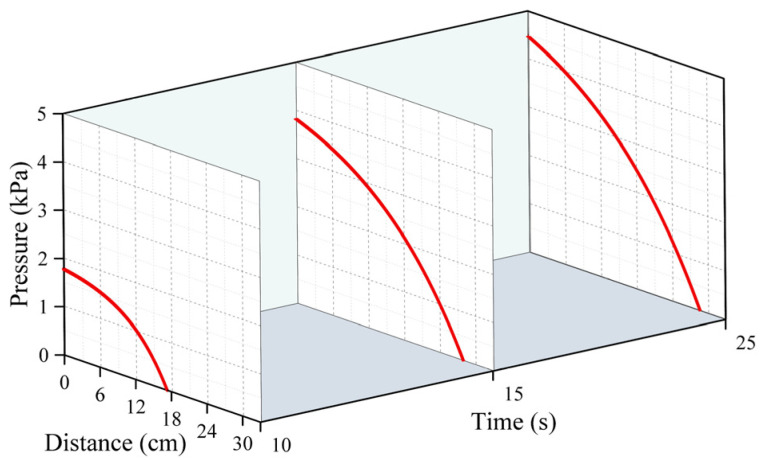
The distribution pattern of pressure at different times when the amount of grouting was 150 g.

**Figure 6:**
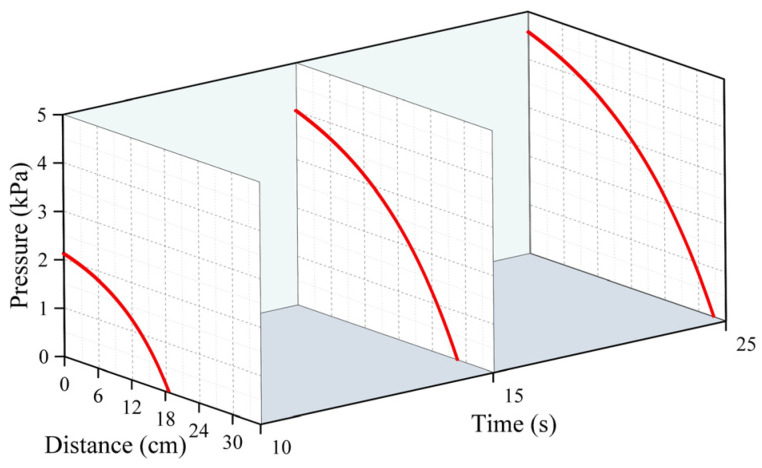
The distribution pattern of pressure at different times when the amount of grouting was 300 g.

**Figure 7:**
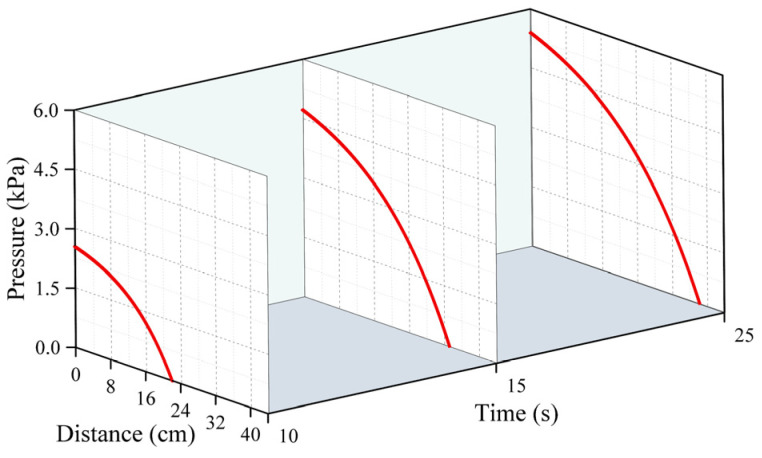
The distribution pattern of pressure at different times when the amount of grouting was 500 g.

Figure 5, Figure 6 and Figure 7 represent the changes in the distribution of the pressure of the polymer slurry during the diffusion process at different moments when the grouting’s mass was 150 g, 300 g, and 500 g, respectively. According to the results of the numerical simulation, the distance of the slurry’s movement stopped increasing when the slurry’s pressure reached zero. As time passed, the radial distance of the polymer slurry’s movement also grew, with the maximum distance reaching 34 cm after 25 s of injection. Additionally, as the slurry’s mass increased, both the pressure and distance of movement of the slurry increased, with the maximum pressure reaching 5.7 kPa when injecting 500 g of slurry. The pressure surrounding the slurry holes decreased in a parabolic manner in the radial direction of diffusion. Over time, the pressure steadily rose in the same spot. The highest pressure of the slurry occurred at the point where the slurry was injected, while the lowest pressure was found at the interface between the slurry and air.

**Figure 8:**
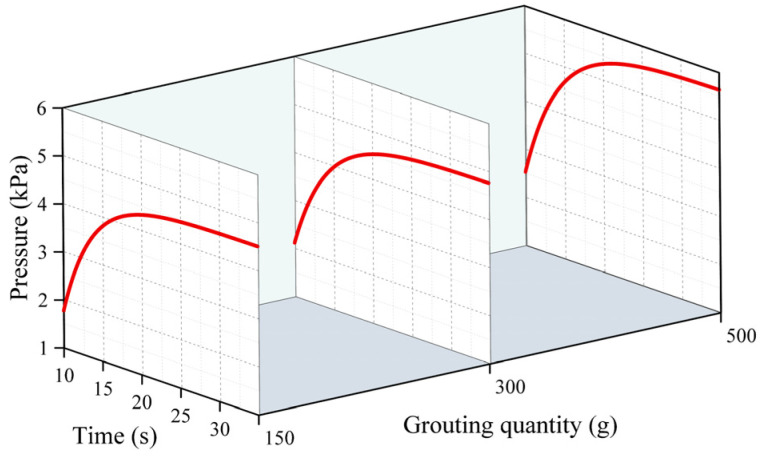
The changes in pressure over time under different quantities of grouting when the movement distance was 5 cm.

**Figure 9:**
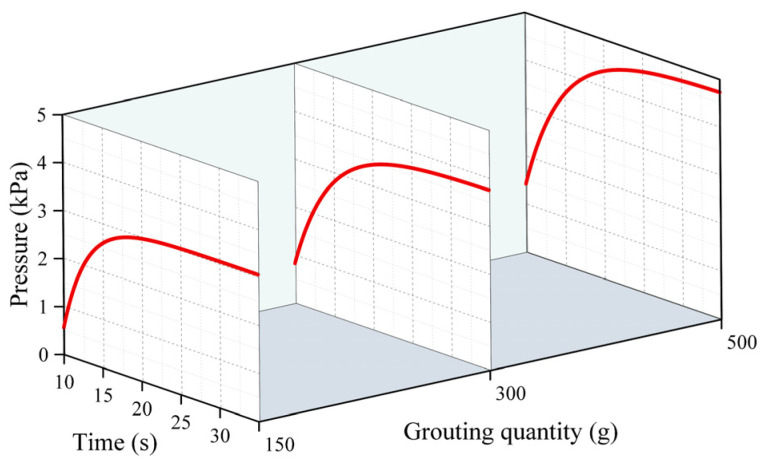
The changes in pressure over time under different quantities of grouting when the movement distance was 15 cm.

**Figure 10:**
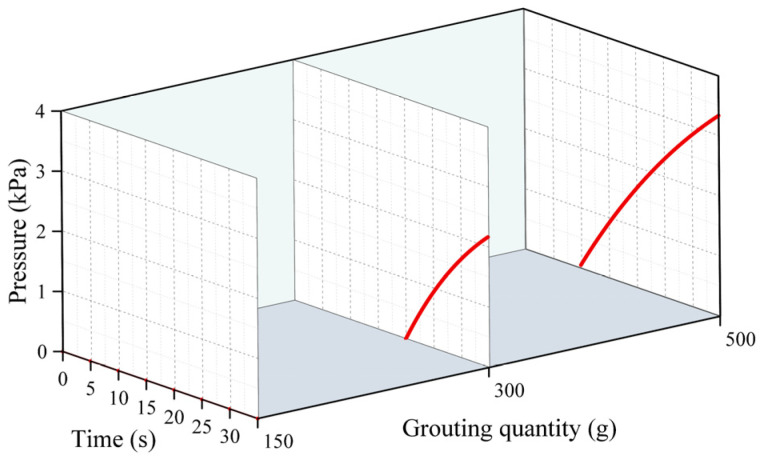
The changes in pressure over time under different quantities of grouting when the movement distance was 30 cm.

Figure 8, Figure 9 and Figure 10 demonstrate the change rule of pressure with time when the slurry’s distance of movement L was 5 cm, 15 cm, and 30 cm under different grouting volumes. Clearly, the pressure rose slowly over time, reaching a plateau after the slurry had spread for 30 s. This is because in the process of the growth in pressure, the polyurethane slurry began to expand and solidify, and when the slurry’s condensation had completed, the solidified body reached a stable state, and the pressure no longer increased. In Figure 10, it can be observed that when the injection volume is 150 g at L = 30 cm, the slurry’s spreading distance never exceeds 30 cm, resulting in a constant pressure of 0 at this location. In Figure 8, it can be observed that the pressure increased as the distance from the grouting hole decreased, reaching a maximum of 5.8 kPa. At positions of 5 cm and 15 cm, the increase in the pressure mainly occurred in the first 20 s, and after 20 s, the pressure gradually tended to stabilize. The distribution of pressure was highest when the amount of grouting was 500 g, exceeding the pressure at 150 g and 300 g.

#### 4.1.2. Experimental Analysis of Pressure Field

Based on the simulated fracture filling test, this study explored the pressure field from the following two perspectives: firstly, we aimed to observe the change in pressure of each monitoring point over the passage of time; secondly, we aimed to analyze how the pressure changed with the change in the movement distance at different time points. This study focused on observing fluctuations in pressure over time at five different monitoring locations located at distances of 5 cm, 10 cm, 15 cm, 20 cm, and 30 cm from the grouting holes.

**Figure 11:**
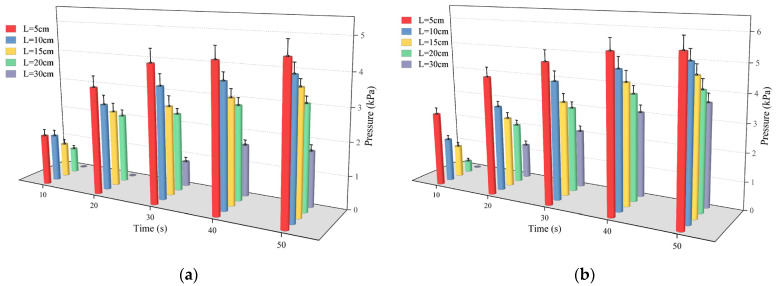
The variation law of pressure over time at different monitoring points when (**a**) the amount of grouting was 300 g and (**b**) the amount of grouting was 500 g.

In Figure 11, the variations in pressure over time at various monitoring locations are depicted for slurry masses of 300 g and 500 g. It was easy to find that at different monitoring points, the pressure increased exponentially with time, and the time of this rise could last for 30 s. When the mass of the slurry was 300 g, the slurry’s pressure increased rapidly from 1.59 kPa to 4.23 kPa, which was nearly threefold. The reason for this trend of the slurry is that the polymer material underwent a chemical reaction in a short period of time which led to the rapid expansion of the material, and then the pressure rose. On the other hand, the higher the mass of the slurry injected into the fissure, the higher the pressure it generated, and when the mass of the injected slurry reached 500 g, the pressure generated was above 4.2 kPa. The proximity of the monitoring point to the grouting hole and the quantity of the injected slurry both contributed to the increase in pressure.

**Figure 12:**
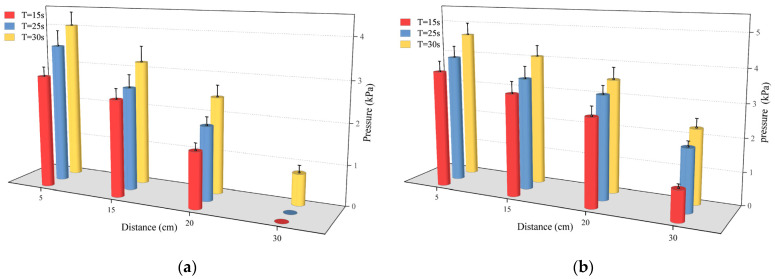
The distribution of pressure at different times when (**a**) the amount of grouting was 300 g and (**b**) the amount of grouting was 500 g.

Figure 12 illustrates that as the movement distance increased, pressure decreased at various time points. Once the slurry had diffused across 20 cm, there was a notable decrease in the pressure gradient, potentially reaching 0. The reason for this phenomenon is related to the driving force of the slurry’s diffusion when the polymer slurry was applied to the fracture, and the expansion produced by the chemical reaction, which increased the volume of the slurry, driving the slurry to move in the radial direction. Due to the minimum expansion force at the junction of the slurry and the air, the slurry was not able to reach stability. Because the expansion force at the junction of the slurry and air was the smallest, the pressure of the slurry after reaching a certain movement distance decreased significantly, even to 0. Furthermore, as the slurry’s mass grew, the total pressure field also rose.

### 4.2. Distribution of Velocity 

**Figure 13:**
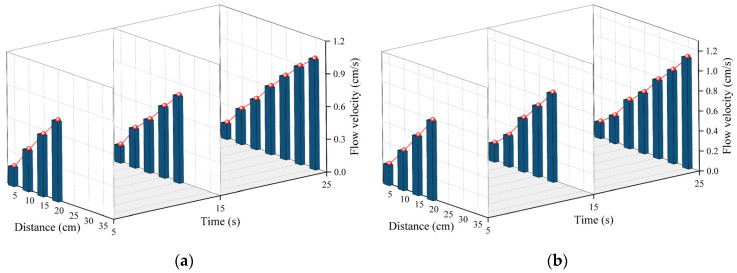
Distribution of velocity at different times when (**a**) the amount of grouting was 150 g and (**b**) the amount of grouting was 300 g.

When the mass of the slurry was 150 g and 300 g, the change rule of the flow velocity of the slurry with movement distance at different moments is shown in Figure 13. The slurry’s flow velocity increased in a linear fashion at 5, 10, and 15 s as the movement distance grew. When the slurry’s mass was 150 g, the flow velocity reached 1.01 cm/s at the 35 cm mark after 25 s, which was seven times faster than the flow velocity at the 5 cm mark at the same time. As mentioned earlier, the driving force of the polymer slurry in the fissure came from the expansion of the slurry at the injection holes at the very beginning, and the flow velocity of the slurry around the injection holes was the lowest because the slurry at the injection holes was subjected to the greatest resistance from the surrounding area. As the spreading distance increased, the resistance of the slurry became less and less until the resistance was minimized at the fluid–air junction, so the flow velocity at the fluid–air junction was the highest.

On the other hand, by comparing the flow’s velocities at the same location at different moments, it can be found that the flow’s velocity decreased slowly with time. In Figure 13b, when the movement distance was 20 cm, the velocity of the flow decreased from 0.8 cm/s to 0.6 cm/s after 25 s of the slurry’s movement. The reason for this phenomenon may be that the driving force for the slurry’s flow decreased as the chemical reaction of the polymer slurry came to an end, and therefore the flow’s velocity decreased along with the time until, eventually, the slurry condensed.

### 4.3. Movement Distance of the Polymer Slurry

#### 4.3.1. Results of the Numerical Calculation of Movement Distance 

**Figure 14:**
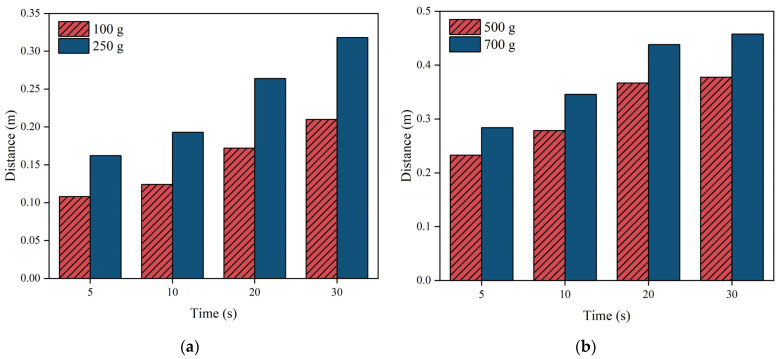
The change in the movement distance with time under different amounts of grouting: (**a**) 100 g and 250 g of grouting, and (**b**) 500 g and 700 g of grouting.

When designing grouting, it is important to take into account how far the slurry will spread. Figure 14 illustrates how the movement distance changed over time for various amounts of grouting. Figure 14 indicates that during the initial 10 s of the slurry’s dispersion, there was not a significant fluctuation in the distance of dispersion. Between 10 to 20 s, there was a notable rise in the diffusion range of the slurry, which then started to decline after 20 s. The reason for this phenomenon may be that in the initial stage of the chemical reaction of polymer slurry, the driving force of the slurry’s flow was not high. After a few seconds, the chemical reaction of the polymer fully activated, and the slurry’s diffusion rate accelerated; after 20 s, the intensity of the chemical reaction of the slurry gradually weakened, and the rate of the slurry’s diffusion then attenuated.

#### 4.3.2. Experimental Analysis of Movement Distance

**Figure 15:**
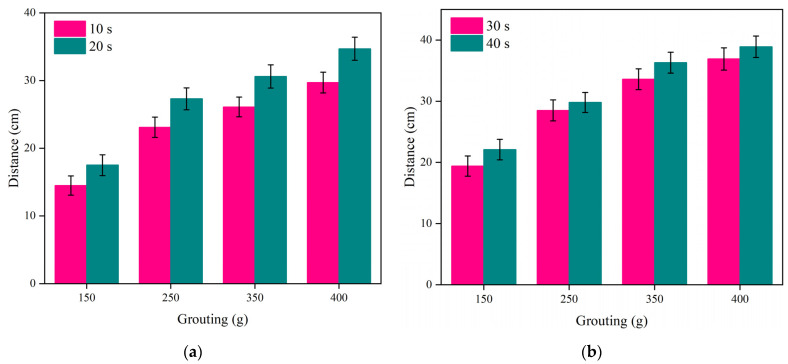
The variation in the movement distance with the amount of slurry at different times: (**a**) 10 s and 20 s; and (**b**) 30 s and 40 s.

Figure 15 illustrates how the movement distance of the polymer slurry changed with the mass of the slurry at various time points. Figure 15a illustrates the expansion of the diffusion range in the slurry from 14.5 cm to 26.1 cm as the slurry’s mass increased from 150 g to 350 g within 10 s. However, the diffusion range only increased by 14% when the slurry’s mass increased from 350 g to 400 g. The reason for this experimental phenomenon may be that within a certain period of time, the rate of chemical reactions occurring inside the slurry increased with an increase in the amount of polymer. Due to space limitations, the rate of the chemical reactions did not increase indefinitely. In a limited crack space, when a certain amount of grouting has been used, continuing to increase the amount of the slurry did not have a significant effect on the growth of the slurry’s permeability rate.

On the other hand, if we compare Figure 11a with Figure 11b, the movement distance of the slurry significantly increased within the first 20 s, but the movement distance of the slurry increased slowly between 30 s and 40 s. The movement distance and diffusion rate of the slurry were positively correlated with the amount of grouting.

**Figure 16:**
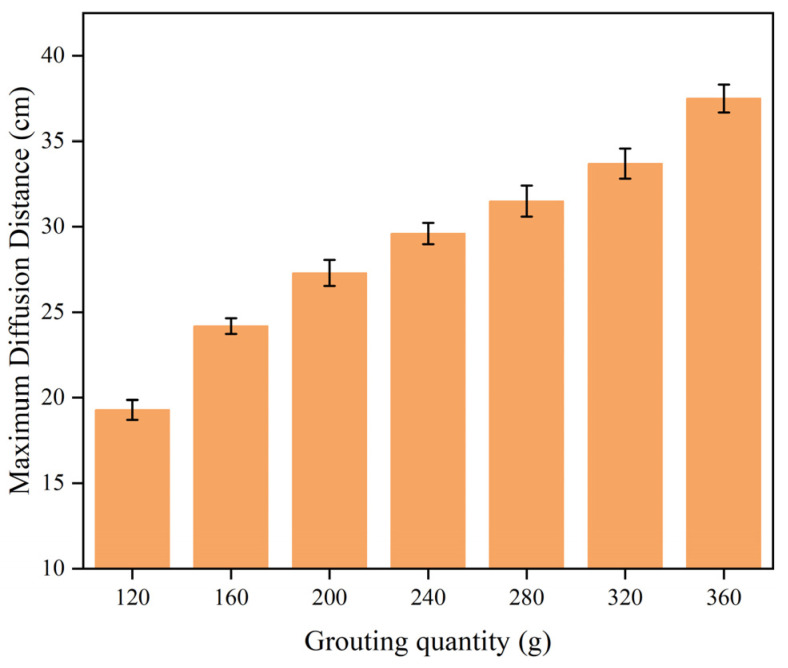
The maximum movement distance varied with the amount of slurry.

As shown in Figure 16, the maximum movement distance of the slurry varied according to the mass of the slurry. Figure 16 shows that as the mass of the polymer slurry increased, the maximum movement distance also increased in a manner resembling a power function. After the injected slurry reached a certain amount, the increase in the maximum movement distance of the slurry was not very significant. Increasing the amount of injected slurry from 120 g to 280 g resulted in a higher maximum movement distance of the slurry, rising from 17.8 cm to 31.5 cm. However, when the mass of the slurry continued to increase to 360 g, the maximum spreading distance of the slurry did not increase significantly: only by 6 cm.

### 4.4. Comparison of Experimental and Numerical Results

**Figure 17:**
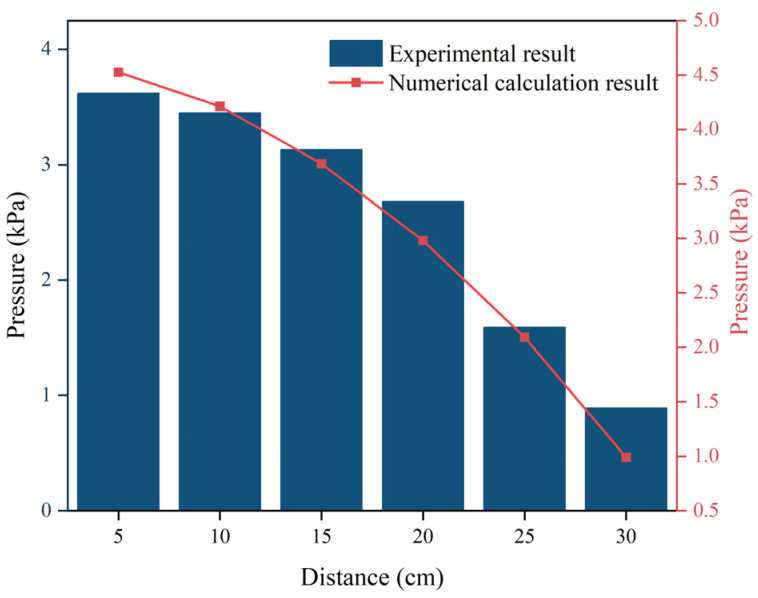
Comparison of the distribution of pressure.

In Figure 17, the distribution of pressure as determined by the numerical model is shown in comparison with the experimental data. As shown in Figure 17, the distribution of pressure predicted by the numerical model showed certain similarities to the experimental data, especially in the trend of variation in the pressure with the movement distance. However, it should be noted that at the same coordinate points, the values of pressure output from the model were higher than those measured in the experiment.

The reasons for some deviation of the numerical simulation’s results from the experimental results may be that, firstly, in order to optimize the engineering applications, the density model proposed in this study was a relatively simplified version for dealing with the important parameter of density. In a real environment, the change rule of density with time is difficult to perfectly resolve during the diffusion of the polymer slurry within the cracks. This, in turn, leads to bias in the numerical calculation. Secondly, in the numerical model, the components of the polymer were presupposed to be idealized mixtures, and it was assumed that the chemical reaction within the polymer occurred simultaneously with the injection of the slurry. However, this is not the case, as the chemical reactions within the polymer slurry start to occur before the slurry is injected into the fracture, which is another reason for the deviation between the numerical simulation’s data and the experimental data.

**Figure 18:**
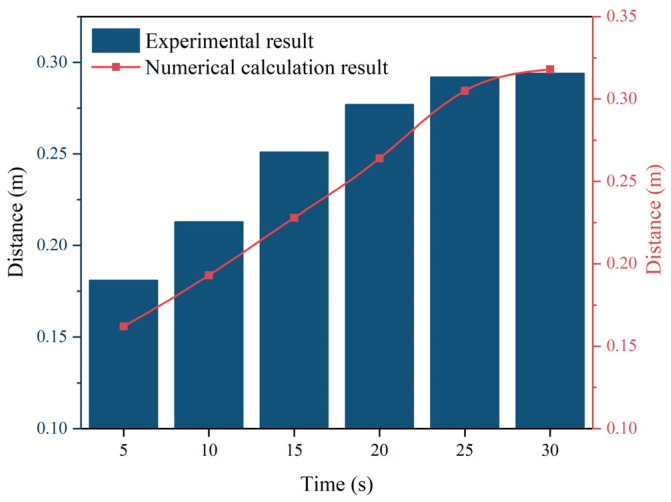
Comparison of the movement distance over time.

In Figure 18, the numerical model’s results are compared with the experimental data for the polymer slurry’s spreading distance. The discrepancy in the spreading distance of the slurry between the numerical simulation and experimental data was most pronounced during the initial 20 s of spreading, with a maximum deviation of 11%. After the slurry’s diffusion reached 30 s, the numerical simulation’s results and the experimental data gradually moved toward the same value, and the deviation was less than 7%. The results of the numerical model for the spreading distance of the polymer slurry were reliable overall.

## 5. Conclusions

This study created a theoretical and numerical modeling system to investigate the diffusion behavior of polymer grouting material in mine fractures at ambient conditions, aiming to understand the diffusion law and to support decision-making regarding grouting points and optimization of volume in mine projects. The indoor simulated test of fissure grouting revealed the variations in the parameters of polymer slurry during diffusion and validated the numerical simulation system. In contrast to Li et al. ’s investigation into the diffusion characteristics of polyurethane within cracks [32], this study contributes to the field by incorporating numerical simulation research on polyurethane’s diffusion. Through the development of a comprehensive theoretical and a numerical model system, the diffusion mechanism of polymer grouting materials at ambient temperature and pressure conditions was elucidated in greater depth. The research’s results showed the following.

- During the diffusion process of polymer slurry, accompanied by a foaming reaction, a large amount of carbon dioxide was generated, which led to the growth and expansion of bubbles. Therefore, the density of the slurry decreased exponentially with the occurrence of a chemical reaction, from about 1.29 g/cm^3^ to 0.073 g/cm^3^ in about half a minute. It was not until the chemical reaction had completed that the density of the polymer could stabilize.
- As the polymer slurry rapidly expanded in a brief timeframe, the pressure of the slurry decreased in a parabolic manner over time. The greater the amount of grouting, the greater the pressure. When the mass of the slurry was 500 g, the maximum pressure of the slurry was up to 5.7 kPa. As the slurry undergoes diffusion movement, the pressure decreases with increasing distance from the slurry injection holes. The location of the slurry’s minimum pressure is observed at the interface between the slurry and air. The slurry’s speed rose steadily as the distance of diffusion increased. Near the grouting hole, the slurry’s speed was low, staying around 0.2 cm/s. The speed was highest at the slurry–air interface, reaching a maximum of 1.1 cm/s.
- The diffusion behavior of polymer slurry in cracks could be divided into three stages: the static pressure injection stage, the slurry’s expansion stage, and the curing stage. The movement distance of polymer slurry was affected by the amount of grouting. With an increase in the amount of grouting, the movement distance of the slurry increased exponentially. When the amount of grouting increased from 120 g to 280 g, the maximum movement distance of the slurry increased by 77%.
- The comparison between the ANSYS FLUENT 2022 R1 simulation’s results and the fissure grouting test’s results revealed a close match in the spreading distance of the slurry, with only a 7% deviation. Additionally, the distribution trend of the pressure field aligned with the test results, validating the numerical simulation method proposed in this study. Although the predictive accuracy of the magnitude of pressure needs to be improved, these studies can still provide theoretical guidance for the application of polymer slurries in underground mine fissure-filling projects.

Through numerical calculations and simulated grouting tests, it is easy and quick to determine the grouting’s volume, the distribution of pressure, and real-time positional changes in the slurry’s flow, and then analyze and predict the location of the grouting holes and provide a theoretical basis for the configuration of the polymer slurry to ensure that the effect of grouting is highly efficient and economical.

## Data Availability

The data presented in this study are available in the article.
